# Molecular characterization of *Cryptosporidium* isolates from humans in Ontario, Canada

**DOI:** 10.1186/s13071-020-04546-9

**Published:** 2021-01-22

**Authors:** Rebecca A. Guy, Christine A. Yanta, Pia K. Muchaal, Marisa A. Rankin, Karine Thivierge, Rachel Lau, Andrea K. Boggild

**Affiliations:** 1 Parasite Biology Unit/Division of Enteric Diseases, National Microbiology Laboratory, Public Health Agency of Canada, 110 Stone Road West, Guelph, ON N1G 3W4 Canada; 2 Centre for Food-borne, Environmental & Zoonotic Infectious Diseases, Public Health Agency of Canada, 370 Woodlawn Road West, Guelph, ON N1H 7M7 Canada; 3 Laboratoire de santé publique du Québec, Institut national de santé publique du Québec, 20045, chemin Sainte-Marie, Sainte-Anne-de-Bellevue, Québec H9X 3R5 Canada; 4 Public Health Ontario Laboratory, Public Health Ontario, Toronto, M5G 1M1 Canada; 5 Tropical Disease Unit, Toronto General Hospital, Toronto, M5G 2C4 Canada; 6 Faculty of Medicine, University of Toronto, Toronto, M5S 1A8 Canada

**Keywords:** Coccidiosis, *Cryptosporidium parvum*, *Cryptosporidium hominis*, *Gp60*, Sanger sequencing, Zoonosis

## Abstract

**Background:**

Cryptosporidiosis is a gastrointestinal disease with global distribution. It has been a reportable disease in Canada since 2000; however, routine molecular surveillance is not conducted. Therefore, sources of contamination are unknown. The aim of this project was to identify species and subtypes of *Cryptosporidium* in clinical cases from Ontario, the largest province in Canada, representing one third of the Canadian population, in order to understand transmission patterns.

**Methods:**

A total of 169 frozen, banked, unpreserved stool specimens that were microscopy positive for *Cryptosporidium* over the period 2008–2017 were characterized using molecular tools. A subset of the 169 specimens were replicate samples from individual cases. DNA was extracted directly from the stool and nested PCR followed by Sanger sequencing was conducted targeting the small subunit ribosomal RNA (SSU) and glycoprotein 60 (*gp60*) genes.

**Results:**

Molecular typing data and limited demographic data were obtained for 129 cases of cryptosporidiosis. Of these cases, 91 (70.5 %) were due to *Cryptosporidium parvum* and 24 (18.6%) were due to *Cryptosporidium hominis*. Mixed infections of *C. parvum* and *C. hominis* occurred in four (3.1%) cases. Five other species observed were *Cryptosporidium ubiquitum* (*n* = 5), *Cryptosporidium felis* (*n* = 2), *Cryptosporidium meleagridis* (*n* = 1), *Cryptosporidium cuniculus* (*n* = 1) and *Cryptosporidium muris* (*n* = 1). Subtyping the *gp60* gene revealed 5 allelic families and 17 subtypes of *C. hominis* and 3 allelic families and 17 subtypes of *C. parvum*. The most frequent subtype of *C. hominis* was IbA10G2 (22.3%) and of *C. parvum* was IIaA15G2R1 (62.4%).

**Conclusions:**

The majority of isolates in this study were *C. parvum*, supporting the notion that zoonotic transmission is the main route of cryptosporidiosis transmission in Ontario. Nonetheless, the observation of *C. hominis* in about a quarter of cases suggests that anthroponotic transmission is also an important contributor to cryptosporidiosis pathogenesis in Ontario.
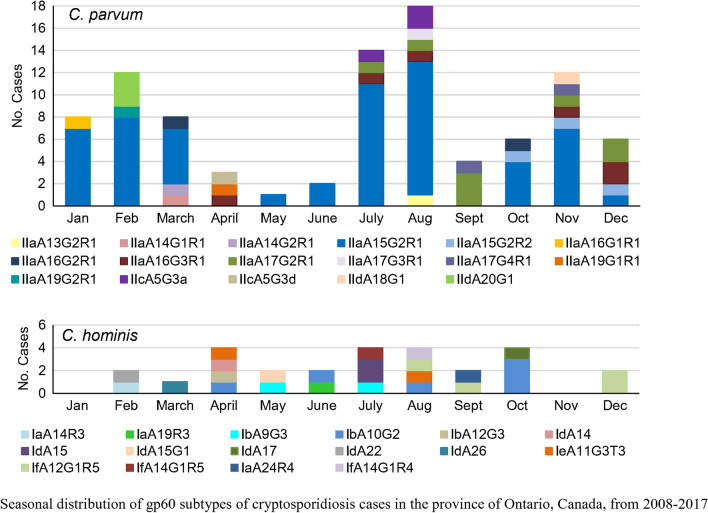

## Background

*Cryptosporidium* is a protozoan parasite that causes gastrointestinal disease worldwide and is a major cause of severe morbidity and mortality in young children in developing countries [1]. In 2016, it was the leading cause of diarrheal mortality in children under 5 years old with 48,000 deaths due to acute infection [2]. *Cryptosporidium* can be transmitted indirectly through ingestion of contaminated food or water, or directly from person to person or animal to person. Infective oocysts are stable in the environment for long periods, are resistant to standard disinfection strategies such as chlorination and iodination, and thus are easily transmitted in water.

Cryptosporidiosis has been a reportable disease in Canada since 2000. Since then, reported annual laboratory-confirmed cases [3] number only 723 on average, but due to the constellation of under-diagnostics and significant under-reporting, estimated annual cases are 35,092 [4]. Higher rates of cryptosporidiosis have been reported in the arctic region of Canada [5–7]. National reporting in the UK, Ireland, France, Sweden and the USA has revealed national and regional differences in species distribution and subtypes of *Cryptosporidium* in humans and high under-ascertainment of this disease [8].

In Canada, as in most countries, national reporting requirements are to the genus level (*Cryptosporidium* spp.); hence, information regarding transmission of sporadic cases through zoonotic or anthroponotic routes is unknown. There are over 38 species of *Cryptosporidium*, 2 of which are major species identified in humans, *Cryptosporidium hominis* and *Cryptosporidium parvum*, and 21 other species of *Cryptosporidium* infect humans less frequently [9]. In developing countries, the human host-adapted *C. hominis* predominates, whereas both *C. parvum* and *C. hominis* are observed in developed countries [8], with a predominance of *C. parvum* in parts of Europe [10–13], Northern USA [14–16] and Canada [7, 17].

Understanding the species and subtypes of disease causing *Cryptosporidium* requires molecular analysis of the parasite. A handful of studies have used molecular typing to identify *Cryptosporidium* species and genotypes in humans in Canada, for source attribution and mitigation of infection. Similar zoonotic *C. parvum gp60* subtypes among humans and cattle were reported in southwestern Ontario [18] and in Prince Edward Island (PEI) [17]. These studies were limited because of the very low number of samples suitable for PCR analysis because in Canada stool is routinely placed into formalin-based fixatives for microscopy analysis of parasites. Some first-line laboratories are moving to molecular diagnosis methods, which may lead to enhanced genotyping when parasite testing is requested. Recently, high rates of *C. parvum* were reported in humans in Quebec [7] with similar zoonotic subtypes as reported in Ontario and PEI [17, 18].

Analysis of 36 endemic cryptosporidiosis cases from 2005–2007 in the Ontario FoodNet Canada sentinel site identified several risk factors for cryptosporidiosis: swimming in a lake or river, consuming municipal drinking water and/or having a family member with a diarrheal illness [19]. Passive surveillance data from 161 Ontario cases with exposure data between 2007 and 2009 determined primary exposures for infection to be associated with animal (47.2%), food (9.3%), person-to-person (11.8%) or water (29.2%) transmission [20]. No laboratory confirmation of species and subtype were available to support source and transmission routes for those studies.

The aim of this study is to better understand reservoirs of infection and transmission dynamics in Ontario. Ontario is the most populous jurisdiction of the ten provinces and three territories in Canada, with a reported 14.7 million people in 2019 [10.25318/1710000901-eng], comprising 38.8% of Canadians. It is home to the largest urban centers in the country, with cities such as Toronto and Mississauga, as well as large regions of agriculture/livestock production, especially in the southwest of the province. Thus, potential for exposure to diverse *Cryptosporidium* species and subtypes exists in Ontario. Annual reported cases of cryptosporidiosis in Ontario, between 2008 and 2017, ranged from 299 to 469 cases [21]^1^.

The objectives of this study were to identify the species and subtypes of *Cryptosporidium* in sporadic cases from a random subset of *Cryptosporidium*-positive human stool samples from 2008–2017, submitted to the provincial reference laboratory in specimen collection containers lacking fixative. Through molecular analysis of the small subunit ribosomal RNA (SSU) and *gp60* gene loci, our aim was to gain a better understanding of potential sources of infection of *Cryptosporidium* in Ontarians.

## Methods

### Samples

Samples used for molecular analysis in this study were surplus unpreserved stool specimens positive for *Cryptosporidium* spp. by routine microscopic fecal examination (including light microscopic examination of iron-haematoxylin stained smears and formalin-ethyl acetate concentrates, as well as auramine-rhodamine fluorescence microscopy) during the period of 2008–2017. These samples were identified and retrieved from the – 20 °C biobank at the Public Health Ontario Laboratory. Demographic information, including age, sex, travel history and clinical symptoms, were obtained, provided by the ordering physician on the standard Public Health Ontario Laboratory test requisition (https://www.publichealthontario.ca/-/media/documents/lab/general-test-requisition.pdf?la=en).

### Ethics

Ethics approval was granted by the Research Ethics Board of Health Canada/Public Health Agency of Canada (Ethics certificate REB 2016-010P). Specimens were transferred to the Public Health Agency of Canada from Public Health Ontario in accordance with a Material Transfer Agreement (MTA 16-033).

### DNA extraction

Frozen fecal samples were stored at – 20 °C. Following thawing, the stool was mixed, and a 0.2 g aliquot of stool was processed using the QIAamp Fast DNA Mini Stool kit (Qiagen, Hilden, Germany). The manufacturers’ instructions were followed with this exception: after incubation in InhibitEx buffer plus proteinase K at 56 °C for 1 h, the samples were subjected to five consecutive cycles of freeze/thaw for 2 min each in liquid nitrogen and in a dry bath at 65 °C. DNA was eluted in two rounds of 50 µl AE buffer and stored at − 20 °C until use.

### Nested PCR (nPCR)

All samples were analyzed at the SSU and *gp60* loci using nested PCR. Platinum Taq (Invitrogen by Life Technologies, Carlsbad, CA, USA) was used as the polymerase and bovine serum albumin, heat shock fraction (Sigma-Aldrich, St Louis, MO, USA) was added at a final concentration of 300 ng/µl to all primary reactions to reduce PCR inhibition. For the SSU assays, primer concentrations used were as previously described [22]. PCR reactions were carried out in 50 µl reactions, and 2 µl of the primary product was used in the secondary PCR. Samples that were negative in repeated SSU nPCR were analyzed using an HSP70 nPCR assay [23]. Part way through the study, screening for *C. parvum* (LIB13 locus) and *C. homini*s (A135 locus) was performed using a multiplex real-time PCR assay [24].

The *gp60* PCR was conducted in 100 µl reactions using species-specific primers for *C. parvum*, *C. hominis* and *Cryptosporidium cuniculus* [25], *Cryptosporidium felis* [26], *Cryptosporidium meleagridis* [27] and *Cryptosporidium ubiquitum* [28]. Amplification was conducted on a Biometra instrument using the cycling conditions in the original articles. Samples were run in duplicate, and nPCR was repeated if the first nPCR was negative. PCR fragments were analyzed using capillary electrophoresis on a QIAxcel DNA Screening cartridge (Qiagen, Hilden, Germany) using the AM420 method. Qiagen’s QX 15bp-3kb Alignment Marker and 50-800 bp size marker were run with the samples.

### Sequencing

Positive samples were purified using the MinElute purification kit (Qiagen, Hilden, Germany) prior to bidirectional Sanger sequencing performed at the CRCHUL sequencing center in Quebec City. Sequence chromatograms were analyzed using BioEdit and MEGA6 programs and on our newly developed SSU and *gp60* typer program called CryptoGenotyper [29, submitted]. Fasta files for each sequence were analyzed using BLAST (Basic Local Alignment Search Tool, NCBI) analysis. Naming of subtypes was based on the scheme described by Xiao [30].

### Nucleotide sequence accession numbers

Unique and rare, partial *gp60* sequences were deposited in GenBank under accession numbers MT953499-MT953508.

### Data analysis

Data including age, sex, travel history and month of primary routine clinical testing were obtained and housed in an encrypted, password-protected Excel file to which only the investigators had access. Analyses were performed to describe the age, sex and seasonal distribution of *Cryptosporidium* species and subtypes. Differences in cases by causative agent (i.e. species of *Cryptosporidium*) were determined using the non-parametric Kruskal-Wallis test. The Fischer’s exact test for significance was applied for the analysis of categorical variables where cell counts were < 5. All statistical analyses were performed using SAS version 9.4. Level of significance was set at *p* < 0.05.

## Results

### Identification of *Cryptosporidium* in clinical specimens

In Ontario, stool specimens for ova and parasite examination are routinely submitted in a fixative solution of sodium acetate, acetic acid and formalin (SAF). Unfixed stool specimens were used in this study and represented only 9.0% (169/1868) of the total number of specimens submitted during the period 2010–2017. From 2010-2017, there were 0% (0/172), 3.3% (7/211), 14.9% (30/202), 7.1% (14/198), 5.0% (13/258), 17.4% (47/270), 9.8% (30/305) and 10.3% (26/252) unpreserved specimens per year, respectively. This study included two specimens from 2008 and one from 2009; however, the total number of specimens for those years was unavailable.

The 169 *Cryptosporidium*-positive human clinical stool specimens analyzed represented 131 cases of cryptosporidiosis diagnosed between 2008 and 2017. Duplicate and triplicate samples were present for 14 cases. Testing of replicates for ten of the cases resulted in the same species and subtypes identified in all replicates per case, for both the SSU and *gp60* genes. The success rates for SSU and *gp60* typing were 99.2% (130/131) and 95.4% (125/131), respectively. The one case not identified using the SSU nPCR was identified as *C. felis* using an HSP70 nPCR.

Of the 131 cases, 129 individual cases had associated demographic and molecular data. There were a variable number of cases for each year: 2008 (*n* = 2), 2009 (*n* = 1), 2011 (*n* = 5), 2012 (*n* = 24), 2013 (*n* = 10), 2014 (*n* = 5), 2015 (*n* = 35), 2016 (*n* = 29) and 2017 (*n* = 19). Among the 129 cases, seven species of *Cryptosporidium* were identified, with *C. parvum* and *C. hominis* representing 92.2% (119/129) of the cases (Table 1). There were four mixed infections of *C. parvum* and *C. hominis* and five other species that occurred at low frequencies (Table 1). A total of 50.4% (*n* = 65) of cases were female and 49.6% (*n* = 64) were male. Among *C. hominis* infections, there was a significant difference (*χ*^2^ = 4.532, df = 1, *P* = 0.0333) in the age distribution of female cases with *C. hominis* infections (median age = 31 years, range = 7–78 years) compared to males (median age = 9 years, range = 1–62). By contrast, no difference (*χ*^2^ = 2.885, df  = 1, *P* = 0.0844) was noted between infections of *C. parvum* in females (median age = 24 years, range 2–87 years) and males (median age = 19.5 years, range = 2–59 years).

**Table 1:** Percent SSU PCR positive species identification of *Cryptosporidium* in 129 female and male cryptosporidiosis cases in Ontario from 2008–2017

*Cryptosporidium* species	Total cases		Females (*n* = 65)	Males (*n* = 64)
	n	%	%	%
*C. parvum*	91	70.5	36.4	34.1
*C. hominis*	24	18.6	9.3	9.3
*C. ubiquitum*	5	3.9	1.6	2.3
*C. felis*	2	1.6	0.0	1.6
*C. meleagridis*	1	0.8	0.8	0.0
*C. cuniculus*	1	0.8	0.0	0.8
*C. muris*	1	0.8	0.8	0.0
*C. parvum/C. hominis*	4	3.1	1.6	1.6

Not included in Table: one case of *C. parvum* and one of *C. hominis* of unknown gender

### Age distribution of *Cryptosporidium* in males and females

**Fig. 1:**
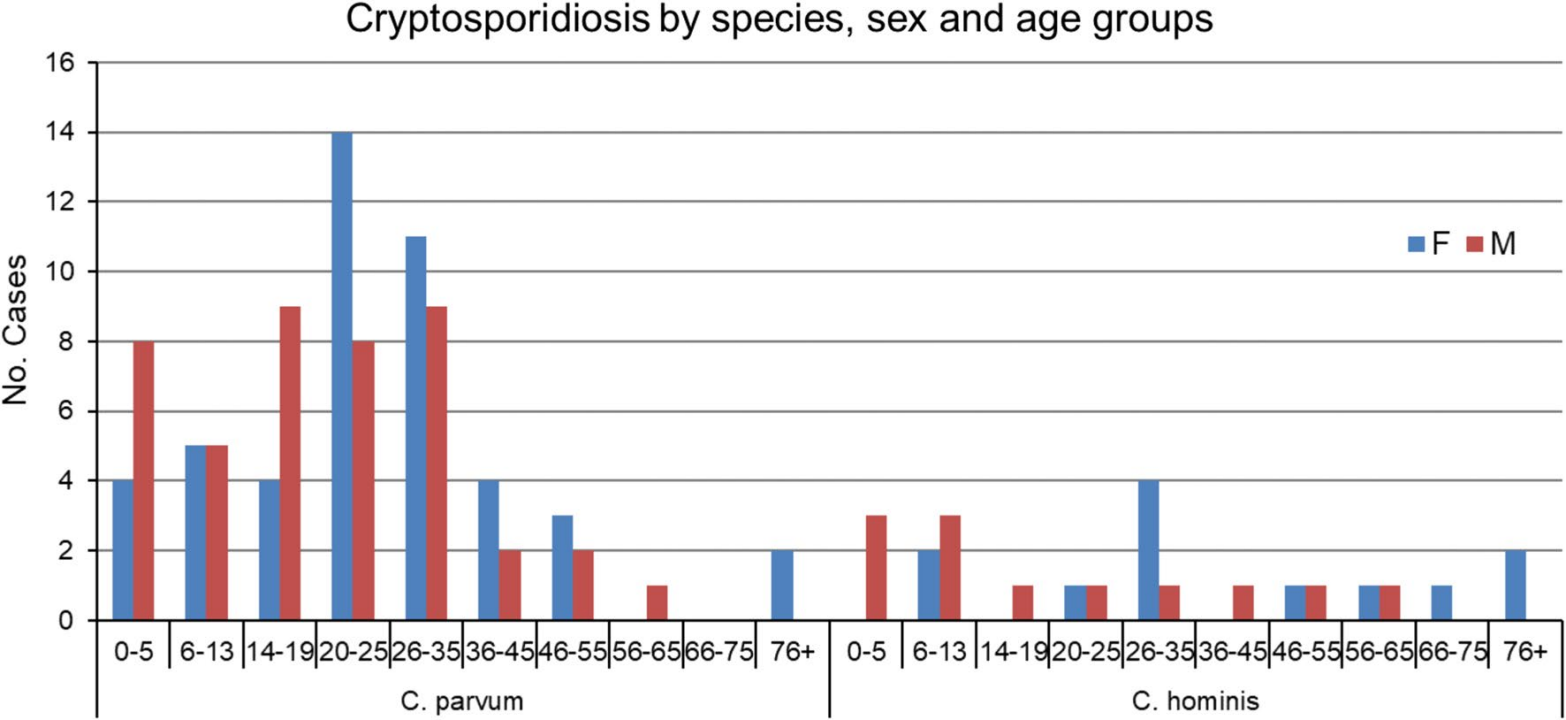
Age and sex distribution of *C. parvum* and *C. hominis* in cryptosporidiosis cases across Ontario from 2008–2017

For all cases of *C. parvum* and *C. hominis*, the median age was 23 years (range = 1–87 years), and the proportion of female and male cases varied by age group (Fig. 1). The median age of female cases was 25 years (range = 2–87 years) compared to 18 years for males (range = 1–62 years). Among children/youth < 20 years of age, there were fewer cases of girls compared to boys, a ratio of 0.58. By contrast, there were 1.6 times as many females compared to males in those aged 20–35 years. The median age of the five *C. ubiquitum* cases was 4 (range = 1–18) years.

### Reported symptoms

Symptom information was available for 62% of cases (82/129). Diarrhea was identified as the only symptom in 36.6% of cases (30/82) and gastroenteritis in 53.7% of cases (44/82). For the purposes of this analysis, it was assumed that gastroenteritis refers to diarrhea or vomiting. In 67.2% of cases (39/58) infected with *C. parvum* subtype IIaA15G2R1, the cases experienced gastroenteritis and/or diarrhea or vomiting.

### Identification of *gp60* subtypes

Subtyping the *gp60* gene revealed a large genetic diversity of *Cryptosporidium* infections among cases of *C. hominis* (5 allelic families, 17 subtypes) and *C. parvum* (3 allelic families, 17 subtypes) (Table 2). Only a single *gp60* subtype was observed in three of the four mixed *C. parvum*/*C. hominis* cases: IbA12G3, IIcA5G3a and IIaA15G2R1. One mixed *C. parvum*/*C. hominis* case had both IfA12G1R4 and IIcA5G3a gp60 subtypes. One *C. parvum* case had mixed IIaA15G2R1 and IIaA16G1R1 subtypes. Two cases of *C. ubiquitum* were subtype XIIb, two cases were XIId, and one case was untypable. One of the two *C. felis* isolates was typed as subtype XIXd-1. The *C. cuniculus* case was subtype VbA38, and the *C. meleagridis* case was IIIeA21G2.

**Table 2:** *Cryptosporidium hominis* and *Cryptosporidium parvum* gp60 subtypes detected in cryptosporidiosis cases in Ontario from 2008-2017

Species (no. subtypes)	Subtype family	Total subtype family		Total subtypes		
		*n*	*%*	Subtype	*n*	*%*
*C. hominis* (*n* = 27)	Ia	4	14.8	IaA14R3	1	3.7
				IaA19R3	1	3.7
				IaA24R4	1	3.7
				IaA25R3	1	3.7
	Ib	9	33.3	IbA9G3	2	7.4
				IbA10G2	6	22.3
				IbA12G3	1	3.7
	Id	7	25.9	IdA14	1	3.7
				IdA15	2	7.4
				IdA15G1	1	3.7
				IdA17	1	3.7
				IdA22	1	3.7
				IdA26	1	3.7
	Ie	2	7.4	IeA11G3T3	2	7.4
	If	5	18.5	IfA12G1R4	1	3.7
				IfA12G1R5	3	11.1
				IfA14G1R5	1	3.7
*C. parvum* (*n* = 94)	IIa	86	91.4	IIaA13G2R1	1	1.1
				IIaA14G1R1	1	1.1
				IIaA14G2R1	1	1.1
				IIaA15G2R1	58	61.6
				IIaA15G2R2	3	3.2
				IIaA16G1R1	1	1.1
				IIaA16G2R1	2	2.1
				IIaA16G3R1	6	6.3
				IIaA17G2R1	8	8.4
				IIaA17G3R1	1	1.1
				IIaA17G4R1	2	2.1
				IIaA19G1R1	1	1.1
				IIaA19G2R1	1	1.1
	IIc	4	4.3	IIcA5G3a	3	3.2
				IIcA5G3d	1	1.1
	IId	4	4.3	IIdA18G1	1	1.1
				IIdA20G1	3	3.2

Two cases with subtype IaA25R3 and IIaA15G2R1, excluded in Table 1 because of a lack of demographic data, are included in this Table. Two cases of *C. parvum* were not typable; one case of mixed *C. hominis*/*C. parvum* had mixed *gp60* subtypes IfA12G1R4 and IIcA5G3a; one case of *C. parvum* contained mixed *gp60* subtypes of IIaA15G2R1 and IIaA16G1

### *gp60* subtypes in travel cases

Only a handful of cases, 3.1% (4/129), were reportedly acquired during travel with known locations for three (75%) of the travel cases. These corresponded to *C. parvum* IIaA19G1R1 acquired on travel to Romania, *C. parvum* IIaA17G2R1 acquired in Taiwan and *C. hominis* IbA10G2 acquired in Lebanon, and the single *C. meleagridis* case was acquired during travel to an undisclosed location.

### Seasonal distribution of *gp60* subtypes

**Fig. 2:**
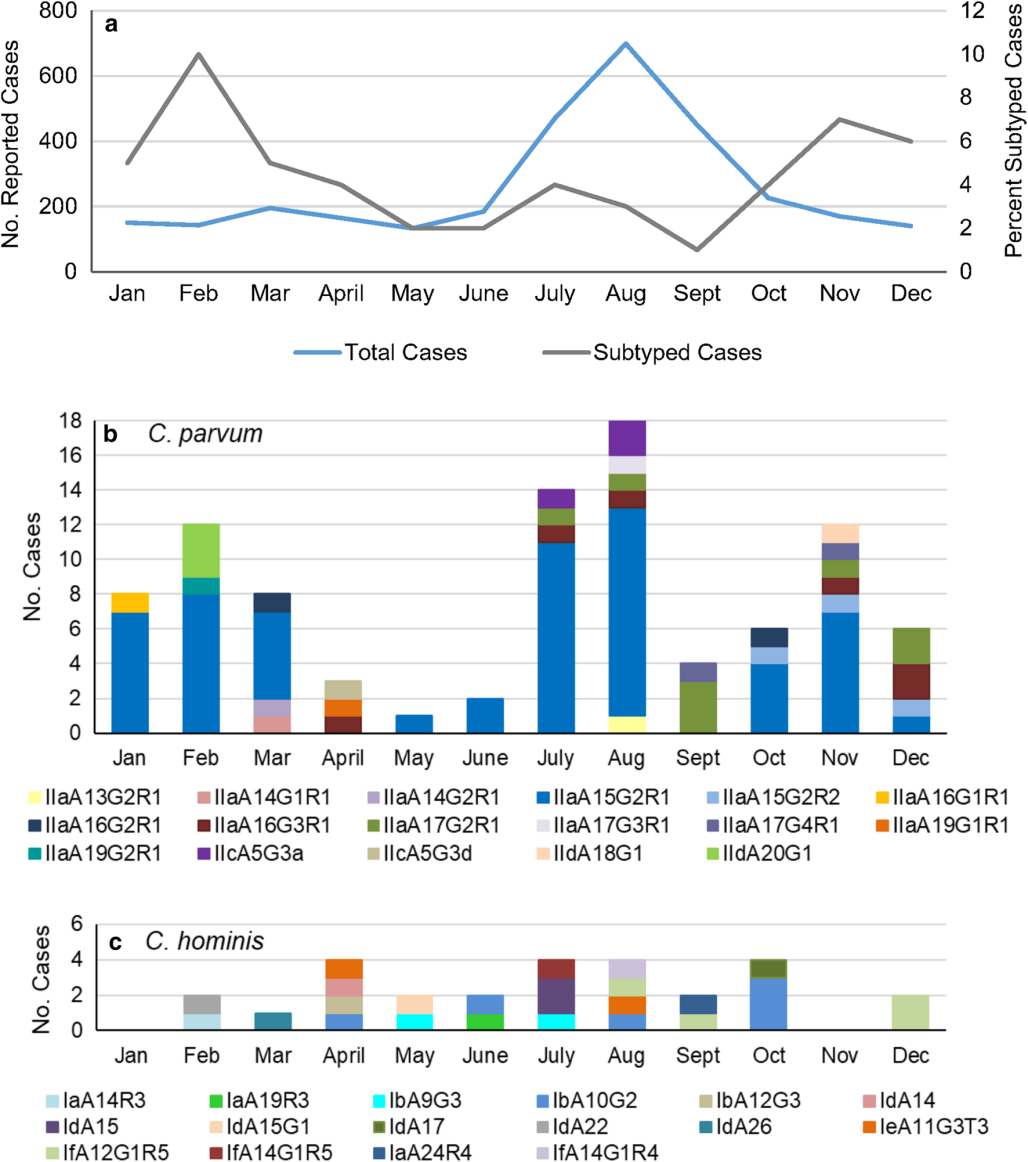
Total cryptosporidiosis cases in Ontario and subtypes of the *gp60* gene of a subset of *Cryptosporidium parvum* and *C. hominis* cases, diagnosed by month for the periods from 2008–2017. **a** Total monthly cases of cryptosporidiosis reported in Ontario versus subtyped cases of *C. parvum and C. hominis* from 2008–2017 (excluding 2010); **b** monthly occurrence of 94 *gp60* subtypes from cases with single and mixed *C. parvum*; **c** monthly occurrence of 27 *gp60* subtypes from cases with single and mixed cases of *C. hominis*

The proportion of samples that we obtained for genotyping from cases in the 9-year study period ranged from 0.01 to 0.1 (1–10%) of the total cases per month reported in Ontario during that period [21]. Cryptosporidiosis cases peaked in the summer, with most of the cases occurring in August (Fig. 2a). A similar trend was observed in individual years (not shown). A different trend was seen when looking at the seasonal distribution of cases with specimens that were subtyped (Fig 2a), with peaks in the winter and fall.

Plotting the subset of samples that were genotyped at the *gp60* locus revealed that *C. parvum* cases were detected throughout the year with a summer peak and a moderate increase during late winter and early spring (Fig. 2b). *Cryptosporidium parvum* IIaA15G2R1 was the most common subtype identified, comprising 62.4% of all *C. parvum* cases, and was the predominant subtype of *C. parvum* and *C. hominis* combined in 6 of the 9 study years (Table 3).

**Table 3:** Proportion of *C. parvum* IIaA15G2R1 *gp60* subtype identified per year in cryptosporidiosis cases from Ontario

Year	Total number reported cases	Total number of *C. parvum* and *C. hominis* cases subtyped	Proportion of cases with the IIaA15G2R1 subtype (%)
2008	338	1	0/1 (0)
2009	309	1	0/1 (0)
2011	301	5	3/5 (60)
2012	299	21	8/21 (38)
2013	309	8	5/8 (63)
2014	362	5	0/5 (0)
2015	387	32	13/32 (41)
2016	429	26	17/26 (65)
2017	391	18	12/18 (67)

**Fig. 3:**
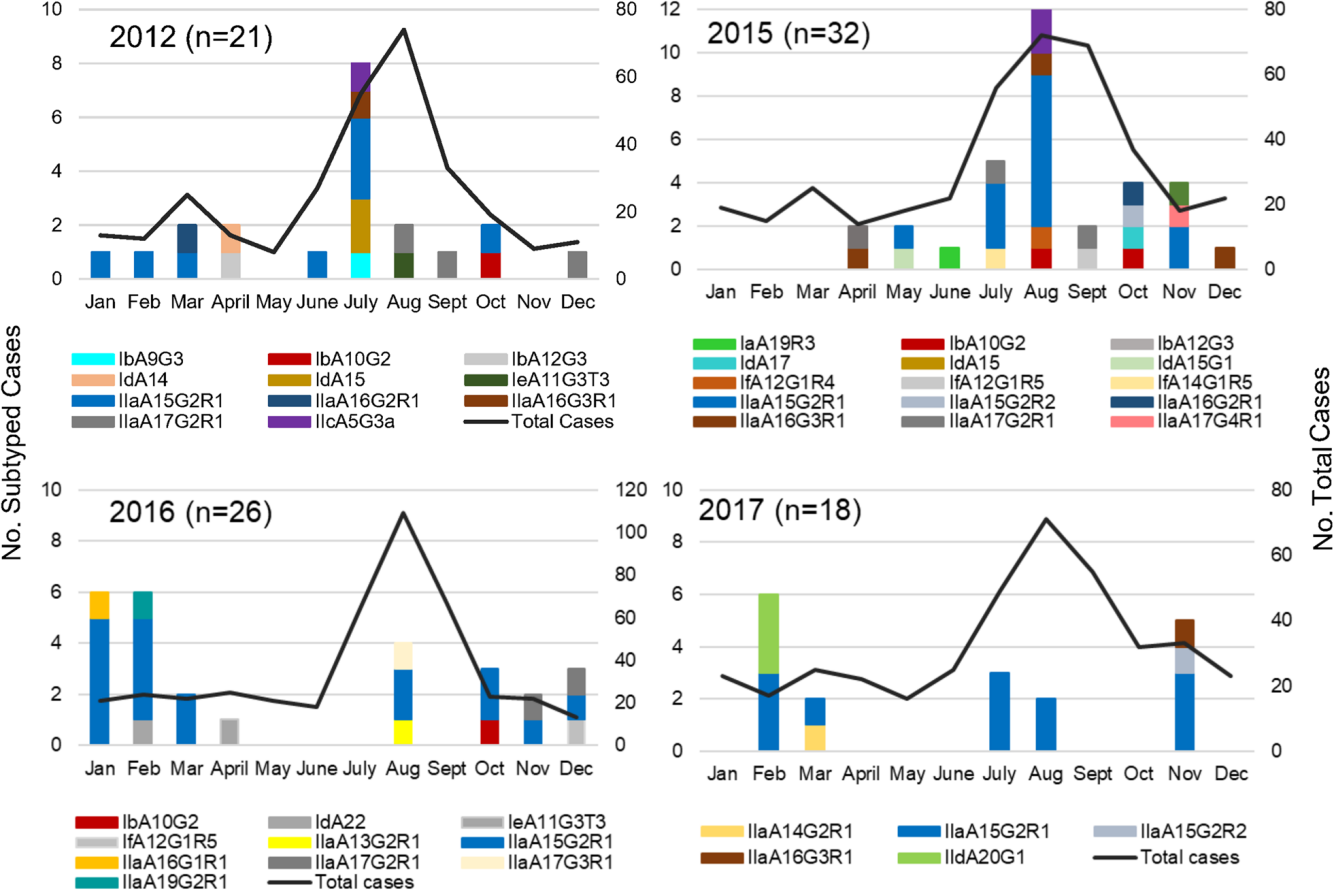
Total cryptosporidiosis cases in Ontario and subtypes of the *gp60* gene of a subset of *Cryptosporidium parvum* and *C. hominis* cases, diagnosed by month, in 2012 and 2015–2017. Total cases are depicted in the line graph and different subtypes depicted in the stacked bars. Subtypes for *C. parvum* and *C. hominis* were combined in each graph, and *n* represents the number of cases of single and mixed *C. parvum* and *C. hominis* cases subtyped for that year

Cases of the IIaA15G2R1 subtype occurred throughout the year, with the exception of April and September, and constituted 12.5% to 87.5% of cryptosporidiosis cases due to *C. parvum and C. hominis* diagnosed each month (Fig. 2b). IIaA17G2R1, the second most frequently observed subtype of *C. parvum,* was only noted in cases that occurred in the second half of the year. *Cryptosporidium hominis* subtypes were observed from February through to October and in December (Fig. 2c). IbA10G2, the most commonly diagnosed subtype of *C. hominis*, occurred in August, September and December. The peak in cases during the summer period was also observed in individual years, 2012 and 2015 (Fig. 3a, b). Higher winter cases were observed in 2016 and 2017 (Fig. 3c, d).

### *gp60* subtypes reported in Canada

A summary of published *gp60* subtypes in humans in Canada (Table 4) shows that of the 204 isolates with *gp60* subtypes reported in the literature, there were 26 subtypes of *C. hominis* and 19 subtypes of *C. parvum*. Twenty-two new subtypes (12 *C. hominis* and 10 *C. parvum*) were identified in the present study that were not previously reported in Canada (Table 4). Subtype IIaA15G2R1 (36.8%) was the most common subtype, followed by IbA10G2 (6.9%) IIaA17G2R1 (5.9%), IIaA16G3R1 (5.4%) and IdA19 (5.4%). Subtyping in two of these studies was performed on outbreak specimens [6, 31].

**Table 4:** Summary of *gp60* subtypes reported in Canadians from five Provinces and one Territory

Subtypes	PEI^h^	Quebec	Nunavut	Ontario	Saskatchewan	BC^i^	Total
*C. hominis*							*71*
**IaA14R3**	–	–	–	**1/27**^f^	–	–	1
IaA19R3	–	–	–	1/27^f^; 1/4^e^	–	–	2
IaA22R3	–	1/11^c^	–	–	–	–	1
IaA23R4	–	–	–	1/4^e^	–	–	1
**IaA24R4**	–	–	–	**1/27**^f^	–	–	1
**IaA25R3**	–	–	–	**1/27**^f^	–	–	1
IbA9G3	–	1/11^c^	–	2/27^f^	–	–	3
IbA10G2	–	6/11^c^	–	6/27^f^	2/11^g^	–	14
**IbA12G3**	–	–	–	**1/27**^f^	–	–	1
IdA13	–	1/14^b^	–	–	–	–	1
IdA14	–	5/14^b^	–	1/27^f^	–	–	6
IdA14G1	–	1/14^b^	–	–	–	–	1
IdA14G2R1	–	1/14^b^	–	–	–	–	1
IdA15	–	5/14^b^	–	2/27^f^	–	–	7
**IdA15G1**	–	–	–	**1/27**^f^	–	–	1
IdA16	–	1/14^b^	–	–	–	–	1
**IdA17**	–	–	–	**1/27**^f^	–	–	1
IdA17G1	–	2/11^c^	–	–	–	–	2
IdA18	–	–	–	–	–	4/4^g^	4
IdA19	–	1/11^c^	–	1/4^e^	9/11^g^	–	11
**IdA22**	–	–	–	**1/27**^f^	–	–	1
**IdA26**	–	–	–	**1/27**^f^	–	–	1
IeA11G3T3	–	–	–	2/27^f^; 1/4^e^	–	–	3
**IfA12G1R4**	–	–	–	**1/27**^f^	–	–	1
**IfA12G1R5**	–	–	–	**3/27**^f^	–	–	3
**IfA14G1R5**	–	–	–	**1/27**^f^	–	–	1
*C. parvum*							*133*
**IIaA13G2R1**	–	–	–	**1/94**^f^	–	–	1
**IIaA14G1R1**	–	–	–	**1/94**^f^	–	–	1
**IIaA14G2R1**	–	–	–	**1/94**^f^	–	–	1
IIaA15R1	–	–	1/7^d^	–	–	–	1
IIaA15G1R1	–	–	2/7^d^	–	–	–	2
IIaA15G2R1	1/5^a^	13/24^c^	3/7^d^	58/94^f^	–	–	75
IIaA15G2R2	–	–	–	3/94^f^; 1/4^e^	–	–	4
IIaA16G1R1	–	–	1/7^d^	1/94^f^	–	–	2
IIaA16G2R1	4/5^a^	3/24^c^	–	2/94^f^	–	–	9
IIaA16G3R1	–	4/24^c^	–	6/94^f^; 1/4^e^	–	–	11
IIaA17G2R1	–	2/24^c^	–	8/94^f^; 2/4^e^	–	–	12
IIaA17G3R1	–	2/24^c^	–	1/94^f^	–	–	3
**IIaA17G4R1**	–	–	–	**2/94**^f^	–	–	2
**IIaA19G1R1**	–	–	–	**1/94**^f^	–	–	1
**IIaA19G2R1**	–	–	–	**1/94**^f^	–	–	1
**IIcA5G3a**	–	–	–	**3/94**^f^	–	–	2
**IIcA5G3d**	–	–	–	**1/94**^f^	–	–	1
**IIdA18G1**	–	–	–	**1/94**^f^	–	–	1
**IIdA20G1**	–	–	–	**3/94**^f^	–	–	3

The following numbers represent the references cited: ^a^ 17; ^b^ 6; ^c^ 7; ^d^ 5; ^e^ 18; ^f^ present study; ^g^ 31. ^h^ PEI = Prince Edward Island; ^i^ BC = British Columbia; subtypes in bold are the first reported occurrence in Canada

## Discussion

This study identified species and subtypes of *Cryptosporidium* on a larger scale than previously reported from Canadians. Based on the total number of samples analyzed at Public Health Ontario each year, the samples tested herein represent a subset ranging from 3.3–17.4% of the submitted specimens per year from 2008–2017. Despite the small sample size, the typing results reveal a diversity of *Cryptosporidium* subtypes detected in Ontario and thus variable potential sources of contamination and transmission routes. Longitudinal molecular surveillance of isolates for species and subtype identification of cryptosporidiosis cases in Europe, Australia and North America [11, 25, 32, 33] highlights the role of molecular testing in surveillance and outbreak detection for prevention and mitigation of cryptosporidiosis.

The distribution of species detected in the Ontario cryptosporidiosis cases mirrors a study in PEI, the province with the highest cattle-to-human ratio in Canada, where 64% of the isolates were *C. parvum* and 37% were *C. hominis* [17], similar to levels reported in Scotland and Sweden [11, 12]. Equal numbers of *C. parvum* and *C. hominis* were observed in an earlier Ontario study [18]. Higher percentages of *C. parvum* (74%) than *C. hominis* (23%) were recently reported from a 2016–2017 study of cryptosporidiosis in Quebec, a province of 8.5 million people that neighbors Ontario. Very high percentages of *C. parvum* were previously reported in Ireland, Denmark and the Northwest USA [10, 13, 15]. Conversely, higher percentages of *C. hominis* occur in much of the USA, Australia and Spain [25, 33, 34]. Longitudinal studies have shown changes in the ratios of *C. parvum/C. hominis* over time in England and Wales, France and The Netherlands [35–38], highlighting the importance of longitudinal surveillance.

Differences in the age distribution of *Cryptosporidium* cases are well documented with a peak in children < 5 years of age [10, 33, 39] or < 9 years of age [11, 32, 35]. This was also observed in this study, in other studies in Ontario [19, 20] and Quebec [7] and in outbreaks in Northern Canada [6]. A peak at 5–10 years of age was reported in PEI [17]. A second peak, in adults 30–39 years old, is also widely reported [11, 38, 39]. We also observed a higher peak of cases in females from 20–35 years old, which could potentially coincide with reproductive years, during which time child rearing could theoretically enhance risk of exposure. This age distribution was not observed in PEI; instead, a second peak was noted among those aged 45–50 years [17].

Differences in *C. parvum* and *C. hominis* geographical distribution exist in some regions, with higher *C. hominis* in urban centers and *C. parvum* in rural regions [32, 40]. Maier et al. [41] reported that people living in rural areas had twice the odds of having *Cryptosporidium* than people living in urban settings; however, molecular data were not available to determine the *Cryptosporidium* spp. in those cases. Information on place of residence for cases in our study was not provided in the dataset.

Cases of *Cryptosporidium* in Ontario peak in the summer months. By contrast, winter and late autumn peaks were evident among the sub-typed cases in addition to the more common summer peak. This bias in ‘seasonal’ distribution may be because some of the specimens provided for subtyping were obtained from a biobank where some unpreserved specimens, of the same case as *Cryptosporidium* positive specimens, were submitted for testing of other enteric pathogens. Insulander et al. [11] reported that people who develop diarrhea after traveling abroad might be more likely to seek medical attention. Travel of many Canadians to the Caribbean and Central America during late fall and winter may in part account for the higher percentage of unpreserved specimens obtained in this study for subtyping during that period. Nonetheless, results of the present study showed a summer peak of cryptosporidiosis due to *C. parvum*, which aligns with summer peaks of cryptosporidiosis cases from passive surveillance data in Ontario [19, 20] and surveillance data from Quebec [7]. This is in contrast to PEI where peak human infections with *C. parvum* were in April–June, corresponding with the time of year of cattle shedding, whereas a late summer/early autumn peak mainly involved *C. hominis* cases [17]. This trend also occurs in England, Wales and Scotland where spring peaks are associated with *C. parvum* and September peaks are associated with *C. hominis* [12, 32, 35, 42].

A study of cryptosporidiosis in cattle (2008–2014) and humans (2009–2015) in southwestern Ontario [43] revealed a peak shedding of *Cryptosporidium* in cattle in late winter/early spring and a peak in human cases of *Cryptosporidium* in mid-summer. Considering environmental conditions, a delay between shedding and environmental exposure through recreational water use was estimated to be a reasonable mechanism of zoonotic transfer of *Cryptosporidium* in the region of Ontario [43]. This may help to explain the summer peak of *C. parvum* cases observed in the present study.

The *gp60* subtype IIaA15G2R1 is the most frequently observed *C. parvum* subtype in humans and calves globally [9] and was the most frequently observed subtype in the current study and in neighboring Quebec [7]. In the USA, the highest rates of cryptosporidiosis are seen in the upper Midwest of the country where predominant subtypes are IIaA15G2R1 in Wisconsin [15] and IIaA16G2R1 in Michigan [14]. In Northern Europe, predominant subtypes are IIaA15G2R1 in Scotland [12], IIaA17G2R1 and IIaA15G2R1 in England and Wales [35, 44] and IIaA18G3R1 in Ireland [10]. In Sweden, IIaA15G2R1 was mainly associated with travel outside the country [11]. The first molecular epidemiology study of cryptosporidiosis in humans and in neonatal dairy calves in Ontario revealed similar *gp60* subtypes in *C. parvum* isolates: 7 subtypes in 44 isolates [18]. Only 11 unpreserved human isolates were available from 2003–2004, and 8 were successfully typed, revealing four *C. parvum* IIa isolates (one IIaA15G2R2, two IIaA17G2R1 and one IIaA16G3R1); all three subtypes were identified in the current study. Interestingly, IIaA15G2R1, one of three predominant subtypes in the Ontario calves, and observed on 10/16 farms in South Western Ontario [18], was not observed among their four human isolates. We observed IIaA15G2R2 in three cases. In PEI, subtype IIaA16G2R1 represented 55% of the cattle isolates, and subtypes IIaA16G3R1 and IIaA15G2R1 represented 22% of the isolates each [17].

A high rate of cryptosporidiosis in Nunavik, Northern Canada, was observed with 15.7% prevalence at a hospital where 108 diarrheal stool samples were analyzed [5]. Seven samples were typed, and all belonged to the *C. parvum* IIa subfamily; three of the seven were the IIaA15G2R1. It is interesting to speculate on the source of this subtype as livestock are not reared in the far north of Canada. Seals may be a potential source of infection as *C. parvum* has been found in seals in Nunavut [45].

The zoonotic IIaA17G2R1 subtype, the second most frequent *C. parvum* subtype in this study, is commonly found in calves and is strongly linked with visits to farms and farm animal contact [44]. It is a predominant subtype in England and Wales [44] and was reported in Ontario and Quebec [7, 18]. IIaA17G2R1 has been found in rodents in Thailand, which suggests rats may be a source of infection [46]. Another zoonotic genotype observed in this study was the IId isolate, a subtype that is host-adapted to small ruminants such as lambs and goats and found in humans [9, 44].

Our data—particularly the finding that *C. parvum* dominated in the samples—support that animal contact may be another important route of transmission in Ontario. Indeed, a Canadian study estimated the annual burden of enteric illness associated with animal contact revealed that *Cryptosporidium* had the highest proportion of illnesses attributable to animal contact of the eight enteric pathogens studied [4].

While potentially zoonotic subtypes predominated in this study, human-host adapted *C. hominis* was observed in 18.6% of cases, and the human host-adapted IIc subfamily of *C. parvum* [9] in three cases, suggesting anthroponotic transmission likely occurred in about a quarter of cases analyzed. IbA10G2, a subtype associated with drinking and recreational water outbreaks in Canada [31], was the most frequently reported *C. hominis* subtype in this study. Thus, recreational water and drinking water are likely sources of this subtype and are known risk factors in Ontario [19]. Trotz-Williams [18], in their study in Ontario, Canada, did not observe the IbA10G2 subtype; instead, they reported four *C. hominis* subtypes (IaA19R3, IaA23R4, IdA19 and IeA11G3T3), two of which (IaA19R3 and IeA11G3T3) were observed in this study. The IdA19 subtype they observed has been associated with swimming pool and drinking water outbreaks elsewhere in Canada [31].

IbA10G2 is the most frequent subtype of *C. hominis* reported in European countries [11, 34, 44]. This subtype was predominant in the USA, until a new subtype, IaA28R4, emerged as the major water-associated subtype in the USA in 2005 [25]. Since 2013, subtype IfA12G1R5 has emerged as the predominant *C. hominis* subtype in sporadic and outbreak cases in the USA [47] and has emerged as one of two dominant subtypes in Western Australia [39]. IfA12G1R5 was observed in three cases in the present study, whereas IaA28R4 has not been reported in Canada to date.

Travel is frequently associated with cases of *C. hominis* in Northern Europe, especially subtypes other than the IbA10G2, in Sweden, England and Wales [11, 48]. Forty percent of unusual cases in England and Wales were associated with travel [49]. While travel was indicated for 4/129 cases in this study, we cannot rule out that other cases were not travel associated, especially in light of previous reports that a quarter of the cryptosporidiosis cases in Ontario are associated with travel [19, 20]. Thus, *C. hominis* infections in Ontario may be acquired during travel or through use of recreational or drinking water.

Two *C. parvum* cases were acquired during travel in this study. One case, acquired during travel to Romania, was IIaA19G1R1, a rare subtype associated with calves, lambs and goats and has been linked to several outbreaks in petting farms with lambs and goats in Norway [52] and a lamb in England [53]. The other case was with IIaA17G2R1.

Other *C. hominis* subtypes observed in the present study were one case of IdA14, two cases of IdA15 and one of IdA15G1. The Id subfamily was the most frequently observed subfamily in an outbreak of cryptosporidiosis in Nunavik, northern Canada [6]. The authors reported five Id subtypes including IdA14 and IdA15 that were observed in this study. A longitudinal study in Western Australia revealed the predominant *C. hominis* subtype was IdA15G1, occurring in 45% of cases [33]. This subtype has been associated with Australian outbreaks in 2007 [50], has a higher prevalence in Aboriginal populations [51] and is the predominant subtype in rural areas of Western Australia [39].

The largest analysis of outbreak data was recently published for England and Wales and demonstrates the power of molecular subtyping for understanding reservoirs of contamination and routes of transmission [54]. The authors demonstrated that IbA10G2 and IIaA15G2R1 predominated for *C. hominis* and *C. parvum*, respectively, in outbreak isolates from England and Wales [54]. These are the major subtypes occurring in Canada; however, we did not have data on outbreak status for the 131 cases in this study. Subtyping of isolates from outbreaks in Canada is available for only a handful of studies, including the large drinking water outbreak in North Battleford, Saskatchewan, in 2001, which affected > 5000 individuals. Both the IdA19 and IbA10G2 subtypes of *C. hominis* were identified in the outbreak cases [31]. The IdA19 has also been identified in a recreational water outbreak in BC [31]. Five Id subtypes were associated with an outbreak in villages in Nunavik in Northern Quebec; the source of the outbreak was not identified [6]. *Cryptosporidium parvum* is implicated in foodborne outbreaks such as an outbreak with subtype IIaA17G1R1 where chopped parsley was the suspected source [11] and subtypes IIaA15G2R1 and IIaA16G1R1 associated with a foodborne outbreak where arugula was the suspected source [11]. IIaA15G2R1 subtype is the dominant subtype of outbreaks in the USA [30].

While *C. parvum* and *C. hominis* comprised 92.2% of the cases in this study, several other species were identified at low frequencies. *Cryptosporidium ubiquitum* was the third most commonly identified species with five cases. Ong et al. [55] were the first to describe *C. ubiquitum* (previously referred to as the cervine genotype) and its association with a swimming pool outbreak in British Columbia (Canada). Trotz-Williams [18] reported a single human case associated with *C. ubiquitum* that was genotyped as the XIIa subfamily, commonly associated with ruminants and described in humans in the UK [28]. The isolates in the current study were both the rodent types XIIb and XIId, subtypes reported in humans and environmental water, including drinking source water in Northeastern USA [28]. Recreational swimming in lakes and rivers is a known risk factor for cryptosporidiosis [19] and may be the route of infection with *C. ubiquitum*.

One of the two *C. felis* cases in this study was typable using the recently developed *C. felis gp60* primers [26] and was subtype XIXd-1. The sequence was homologous with the Swec 053 isolate (GenBank: MH240831.1) from a Swedish traveler to India and clusters closely with 17 other human isolates of *C. felis*, mainly from the UK and Swedish travel cases. This cluster of cases is very conserved and distinct from other *C. felis gp60* clusters [26]. To date, 17/18 reported cases were in males (median age, 37 years) [26]; the case in this study was a male in his 30s.

*Cryptosporidium meleagridis*, a bird-associated species, is also responsible for about 10% of cryptosporidiosis cases globally, likely through zoonotic and anthroponotic transmission [56]. We observed one *C. meleagridis* case with the IIIeA21G2R1 subtype in a female who likely acquired the infection during travel to an undisclosed location. The IIIe subtype has been reported in Swedish travelers to Asia [11], rodents and chickens in Asia [57, 58] and red partridges in Slovakia [59]. Two GenBank submissions of IIIeA21G2R1 (accessed in September 2020) were from a Swedish traveler to Indonesia and a chicken in China (GenBank: KU852728.1 and KJ672091.1).

*Cryptosporidium meleagridis*, *C. canis*, *C. felis* and *C. muris* are more common in developing countries [9] and frequently reported in individuals with HIV and other immunodeficiencies [36, 58] and in immunocompromised individuals [36, 49, 60]. Data regarding immune status were unavailable for the *C. meleagridis* case, two *C. felis* cases and the *C. muris* case detected in this study.

A limitation of this study was the small sample sizes per year and the random selection of isolates characterized. Additionally, not every stool specimen submitted to Public Health Ontario has a corresponding unpreserved specimen, and we cannot exclude the possibility of differential submission patterns over time. Significant under-reporting of symptoms and presentation bias may also influence the types of patients for whom a stool sample is even ordered. Important clinical data—including intercurrent immunosuppression, due to HIV, for example, as well as chronicity of symptoms—are almost universally missing from standard laboratory test requisitions. Thus, the cases represented in this analysis may lack external validity to all patients with cryptosporidiosis in the province of Ontario, and our comparisons may be skewed accordingly.

## Conclusions

This study presents the largest number of cases typed to date in Canada and demonstrates a wide variety of *Cryptosporidium* subtypes present in Ontario cases. As only a fraction of yearly specimens (3–17%) was tested, this study provides an estimate of the potential sources and transmission routes. This study supports previous epidemiological analyses in Ontario in showing that bovine isolates are potentially a large cause of cryptosporidiosis in Ontario [19, 20]. Differences may also occur through changes in patterns of infection over time such as the emergence of the IaA28R4 subtype as the major isolate associated with USA recreational water outbreaks when IbA10G2 predominated prior to 2006. National surveillance programs such as in the UK or the USA CryptoNet program enable detection of changes over time that may provide mitigation and prevention strategies [25, 32]. Routine national molecular surveillance of cryptosporidiosis is more feasible now as there has been a switch to molecular testing in many primary laboratories in Ontario [61] and in other parts of Canada; thus, unpreserved specimens would be more readily available for molecular analyses.

While *gp60* is the most widely used marker globally for subtyping *Cryptosporidium,* further subtyping is required to discriminate *gp60* subtypes, such as the common IIaA15G2R1. Numerous other markers have been identified; however, additional markers will provide greater discrimination of sources of IIaA15G2R1 and other subtypes in humans. Research on whole-genome sequence (WGS) comparisons of diverse human and animal isolates is ongoing, and more WGS data are needed to support identification of new markers to provide robust discrimination between sources, identify risk factors and elucidate patterns of transmission [62]. Ultimately, a harmonized multi-locus typing scheme would provide better analysis of transmission not only in Europe as proposed [63] but also globally.

## Data Availability

The datasets generated during and/or analysed during the current study are not publicly available due to privacy restrictions and ethical considerations but are available from the corresponding author on reasonable request.

## Abbreviations

SSU rRNA: Small subunit ribosomal ribonucleic acid
*gp60*: Glycoprotein 60
nPCR: Nested polymerase chain reaction
PEI: Prince Edward Island
BC: British Columbia
WGS: Whole genome sequencing

## Footnotes

1 Cases reported in Ontario include laboratory-confirmed cases and epidemiological cases linked to a laboratory-confirmed case.
